# Supplementary material for: Interplay between disorder and electronic correlations in compositionally complex alloys
Source: Nat Commun. 2024 Sep 12;15:7983. doi: 10.1038/s41467-024-52349-8 (PMC11393320; doi:10.1038/s41467-024-52349-8)
Supplement: Supplementary file 1 — Supplementary Information [file 41467_2024_52349_MOESM1_ESM.pdf]

Supplementary Information for Interplay between disorder and electronic correlations in compositionally complex alloys

David Redka[1,2], Saleem Ayaz Khan[1], Edoardo Martino[3], Xavier Mettan[3], Luka Ciric[3], Davor Tolj[3], Trpimir Ivšić[3,†], Andreas Held[4], Marco Caputo[5], Eduardo Bonini Guedes[5], Vladimir N. Strocov[5], Igor Di Marco[6,7], Hubert Ebert[4], Heinz P. Huber[1,2,*], J. Hugo Dil[3,5], László Forró[3,8] & Ján Minár[1,**]

[1]*New Technologies Research Center, University of West Bohemia, Plzen CZ-30100, Czech Republic*
[2]*Department of Applied Sciences and Mechatronics, Munich University of Applied Sciences HM, Munich DE-80335, Germany*
[3]*Institute of Physics, École Polytechnique Fédérale de Lausanne, Lausanne CH-1015, Switzerland*
[4]*Department of Chemistry, Ludwig-Maximilians-University Munich, Munich DE-81377, Germany*
[5]*Photon Science Division, Paul Scherrer Institut, Villigen CH-5232, Switzerland*
[6]*Institute of Physics, Nicolaus Copernicus University, Toruń PL-87-100, Poland*
[7]*Department of Physics and Astronomy, Uppsala University, Uppsala SE-75120, Sweden*
[8]*Stavropoulos Center for Complex Quantum Matter, Department of Physics and Astronomy, University of Notre Dame, Notre Dame IN 46556, USA*
†New address: Department of Physical Chemistry, Ruđer Bošković Institute, Zagreb HR-10000, Croatia
*Corresponding authors: *heinz.huber@hm.edu; ** jminar@ntc.zcu.cz;*

Supplementary Note 1: Photoelectron energy distribution curves

Supplementary Fig. 1(a) illustrates the photoelectron energy distribution curves (EDC) of the CrMnFeCoNi HEA containing elements, as measured by resonant photoemission spectroscopy (ResPES). For enhanced visualization, the EDCs are presented as stacked profiles and normalized to their respective maximum. The element specific X-ray absorption spectroscopy (XAS) L_3 maximum is indicated by an arrow. The photon energy (E_{ph}) increment is constant 1 eV. For Cr, there is no shift of the EDC peaks, indicating that there is no transition from the radiationless ResPES regime (constant binding energy E_{B}) to the resonant Auger regime (constant kinetic energy, E_{k}). Accordingly, the EDCs represent the VB (see main manuscript). The situation is somewhat more complicated for Mn, where a clear resonance effect can be seen in the XAS maximum at a E_{B} of 3.6 eV. The slight shoulder at 7.5 eV, if followed towards higher photon energies, can be traced to a small contribution of Auger signals, as seen in the constant kinetic energy behavior. However, in the region distant from the XAS L_3 maximum (lower and higher E_{ph}), the VB seems to dominate with a constant E_{B} behavior. For Fe, Co, and Ni, it becomes apparent that above the corresponding XAS L_3 maximum, there is a transition from constant E_{B} to constant E_{k} of the measured EDC peaks. Consequently, the maxima positions of the EDCs are to be interpreted as Auger peaks (see main manuscript). The shoulders in the XAS maximum towards lower binding energies can be generally assigned to a VB component in the EDCs with constant E_{B} behavior, particularly in EDC curves for photon energies far away from the XAS absorption edge. This is also demonstrated in Supplementary Figure 1(b), which depicts the EDCs (not normalized, but in arbitrary units) for the XAS L_3 maximum and 3 eV below and above it, respectively. Despite the prominent signal at the absorption maximum, it is evident that the VB components at low E_{B} exhibit approximately the same intensity and remain at the same position, distinct from the absorption maximum.

Supplementary Note 2: Ground state calculations of CrMnFeCoNi

Ab initio calculations on the CrMnFeCoNi high-entropy alloy were performed within the fully relativistic spin polarized multiple scattering KKR Green's function method[1] using the Munich SPR-KKR code[2]. Exchange correlation effects were taken into account with the LSDA exchange correlation functional in the parametrization of Vosko, Wilk, and Nusair[3]. The chemical composition of the disordered solid solution was calculated within the framework of the coherent potential approximation (CPA)[4,5]. In the calculations we employed a multipole expansion of the Green's function with a cutoff of $l_{\max} = 3$. A dense grid of 5000 k-points in the irreducible part of the Brillouin zone was used and a low Broyden mixing parameter of 0.05 was applied in the self-consistency cycle. The paramagnetic state, for temperatures above $T_C \approx 20$ K,[6] was calculated within the disordered local momentum approach (DLM)[7,8] with fixed opposite magnetic moments for each element. A fcc crystal structure (Fm-3m) with an lattice constant of 3.5991 Å,[9] corresponding to the experimentally determined room temperature value, was used for the CrMnFeCoNi high-entropy alloy. Taking electron correlations for strongly correlated metals (as it is the case for 3d transition metals) into account, the dynamical mean field theory (DMFT) formalism was applied by means of the FLEX (fluctuation exchange) algorithm[10]. Hereby an element specific on-site Coulomb interaction U and a constant Hund's exchange parameter $J = 0.94$ eV, was considered, with values known to reproduce experimental data for pure elements accurately. These where for Cr, Mn, Fe, Co, Ni equal to 2.0 eV[11], 3.0 eV[12], 1.5 eV, 2.5 eV and 3.0 eV[13], respectively. The element and band-resolved pDOS are displayed in Supplementary Fig. 2 for the LDA and LDA+DMFT case.

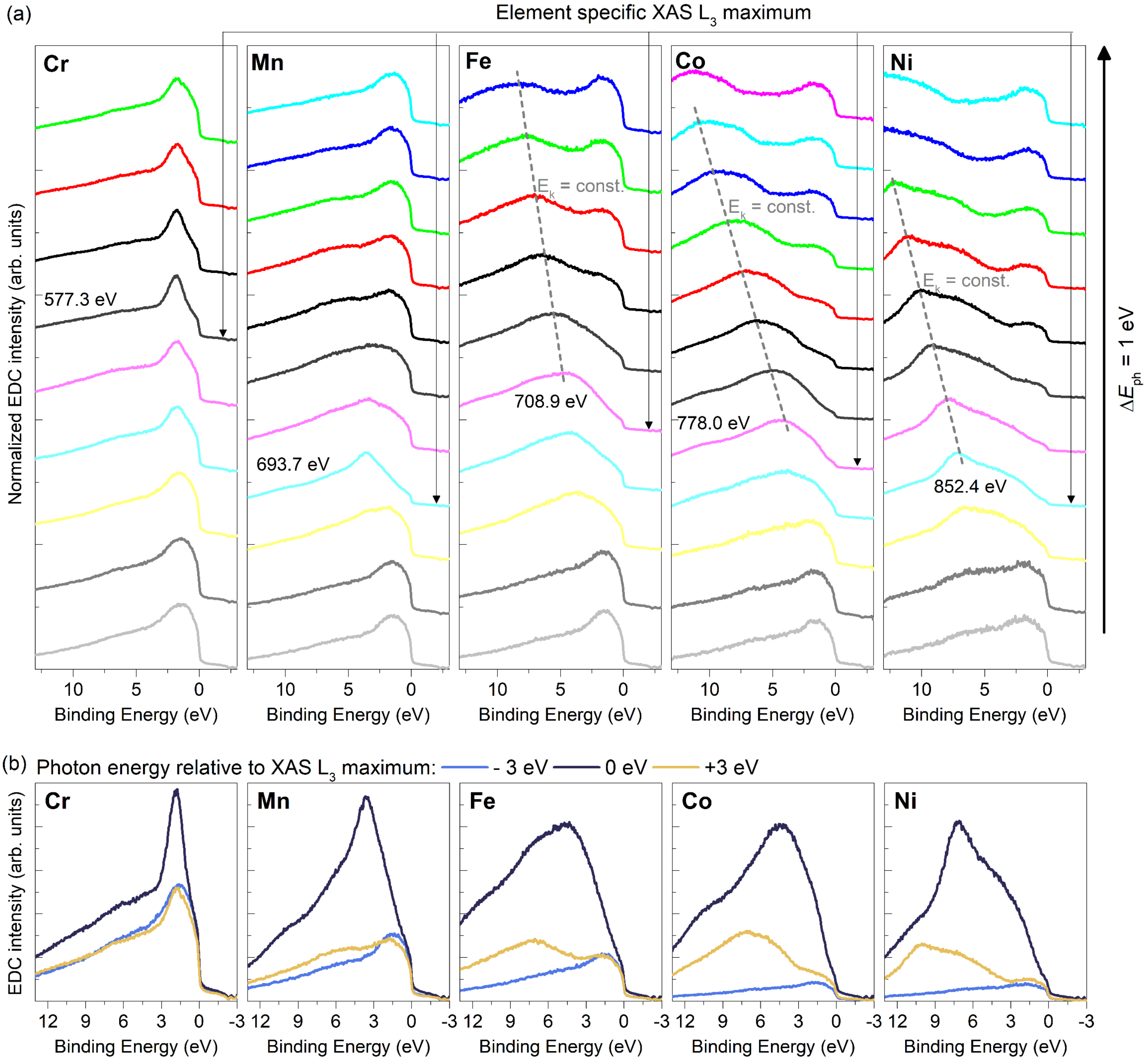

Supplementary Fig. 1 Photoelectron energy distribution curves (EDC) around element specific X-ray absorption spectroscopy (XAS) L_3 edge of CrMnFeCoNi alloy, measured by resonant photoemission spectroscopy (ResPES). (a) The EDCs are presented as stacked profiles and normalized to their respective maxima, with the XAS maximum indicated by an arrow and value given next to the EDC. Photon energy (E_{ph}) increment is consistently 1 eV (from lowest to highest offset). The constant kinetic energy (E_k) behavior of the EDC maxima are highlighted in gray for Fe, Co and Ni, identifying the resonant Auger origin of these peaks. (b) EDCs (in arbitrary units) for measured element specific XAS L_3 maximum (black), 3 eV below (blue) and above (yellow), highlighting VB components at low E_B.

For the resulting paramagnetic ground state within the LDA calculations, the local magnetic moments are 1.29 μ_B for Mn and 1.66 μ_B for Fe. Cr, Co, and Ni do not exhibit significant local magnetic moments, as it was previously observed elsewhere[14]. When applying the LDA+DMFT approach, Cr and Ni maintained negligible local magnetic spin moments. Fe's local magnetic moment was consistently around ±1.66 μ_B across opposite spin channels. Mn displayed a slight asymmetry between spin channels, with values of -1.07 μ_B and 1.08 μ_B, respectively. For the two types of Co atoms, which initially had opposite magnetic moments, a slight asymmetry is observed in the DLM calculation. One type has a magnetic moment of zero, while the other type converges with a low magnetic moment of -0.03 μB. The overall magnetic moment for the LDA+DMFT calculation achieved a nearly balanced state, with a total magnetic spin moment of -0.003 μ_B. Regarding the electronic structure, the Fermi energies determined for both LDA and LDA+DMFT calculations were 0.729 Ry and 0.735 Ry, respectively. In both cases, a consistent total of 8 electrons per atom was maintained, attributable to the 20% elemental mixture within the CPA framework.

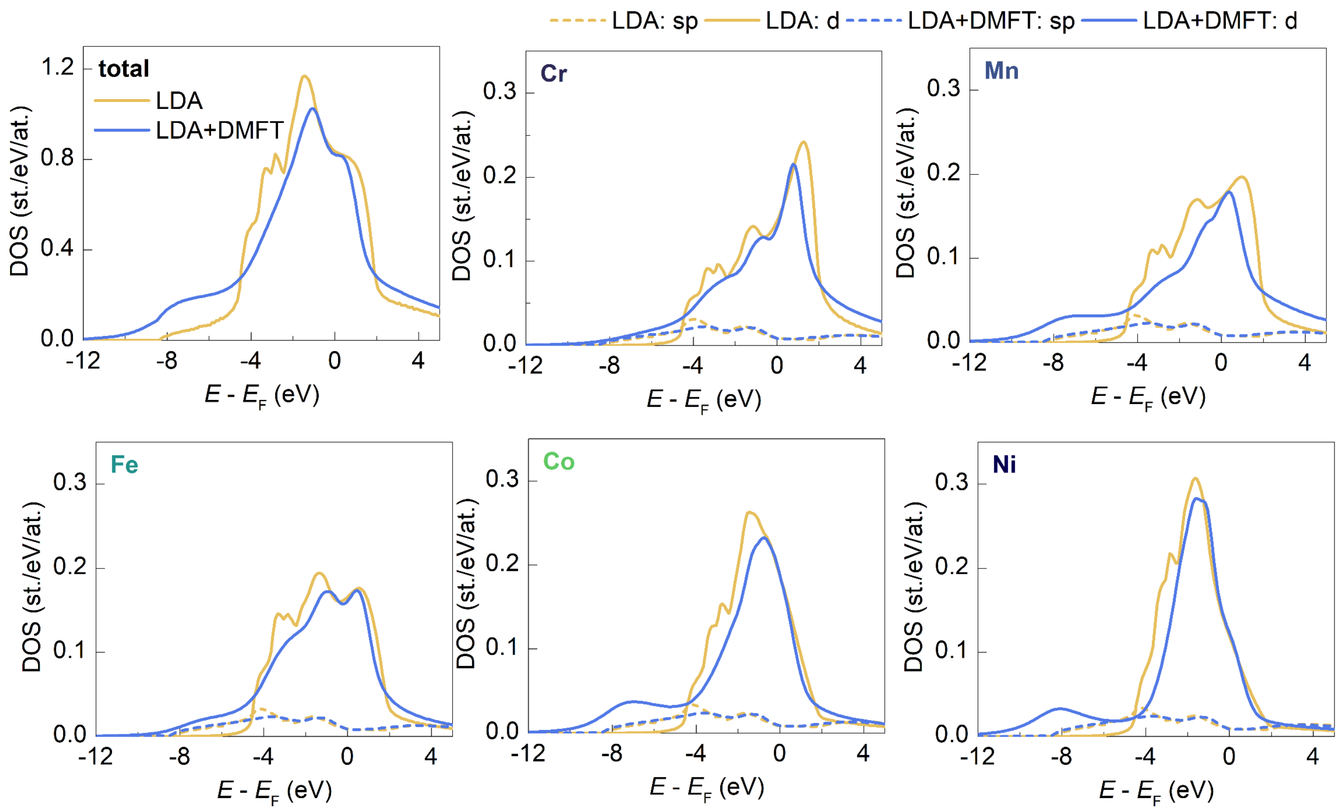

Supplementary Fig. 2 Total and element-resolved partial Density of States (DOS) for CrMnFeCoNi, comparing results from LDA and LDA+DMFT. The total DOS is shown in yellow for LDA and in blue for LDA+DMFT. For the element-resolved partial DOS, both element and band contributions are depicted, with dashed lines representing the *sp*-band and solid lines representing the *d*-band. The coloring scheme remains consistent: yellow for LDA and blue for LDA+DMFT.

Supplementary Note 3: Influence of Hund coupling *J* on electronic structure

The variation of the Hund exchange coupling J across the 3d elemental series (from Cr to Ni) is typically around 0.1 eV as derived by constrained random-phase approximation calculations[15]. In CrMnFeCoNi, local orbital differences might increase this variation by 50 %[10]. However, there are two main reasons why this variation is not fully meaningful in our case. Firstly, the HEA has a different crystal structure, number of neighbors and screening effects compared to some of the pure elements (Cr, Fe, Co). Secondly, a change of J of about 0.1 or 0.2 eV will have no visible effect on our calculations. This is due to the particular computational scheme implanted within SPRKKR, where the DMFT self-energy acts as a correction to a spin-polarized DFT solution. In this approach, which is the most common way to apply DFT+DMFT to magnetic materials, the major effect associated to J, i.e. the renormalization of the exchange splitting, is completely canceled by the double-counting term (see e.g. Ref.[16] for the analysis of the consequences on the effective magnetic coupling).

To substantiate this argument, we performed calculations using the SPRKKR package, focusing on the influence of J variations in ferromagnetic (FM) Ni, paramagnetic (DLM) FeNi, and our CrMnFeCoNi HEA, with U values as specified above.

For FM Ni, a strong variation of J from 0.5 eV to 1.3 eV does not significantly impact the spectral weight distribution of the d-bands near the Fermi level (E-E_F between -5 eV and 0 eV). This may be seen in Supplementary Fig. 3(a) and (b). The main effect is a slight shift of the split-off satellites. For the majority (up) spin channel, the satellite shifts from -8.2 eV to -8.7 eV as J increases. This corresponds to a shift of $\Delta P_S/\Delta J = -0.6$ (P_S is the position of satellite peak). For the minority (down) spin channel, no satellites are observed, only shoulder-like features between -5 eV and -10 eV, which are slightly attenuated by an increased J.

For FeNi, varying J from 0.6 eV to 1.2 eV results in no significant changes in the electronic spectra, as can be seen in the total density of states (DOS) in Supplementary Fig. 3(c). Solely minor influences are observed on the shoulders around -8 eV, but the overall spectral width and *d*-band positions remain unchanged, similar to the minority spin channel of FM Ni.

For CrMnFeCoNi, varying J shows also negligible effects on the electronic structure. Results are depicted in Supplementary Fig. 3(d). The DOS peak position at -1 eV remains constant for all J values, with only a slight decrease in the DOS from 2.01 states/eV/atom to 1.97 as J increases. The DOS at the Fermi level remains unaffected, and the spectral width of the *d*-band

does not change. However, there is a minor shift of the spectral weight towards higher binding energies, visible from the flanks of the d-band, which shift approximately with $\Delta P_f/\Delta J = -0.3$ (P_f is the position of the d-band flanks).

In contrast, the variation of the Hubbard parameter U has a much more pronounced effect, as may be seen in Supplementary Note 4 and the main manuscript. For instance, increasing U from 3 eV to 4 eV for Ni in CrMnFeCoNi results in a direct and isolated shift of the Ni satellite from -8 eV to -10 eV, corresponding to $\Delta P_S/\Delta U = -2$. Given that J varies by about 0.1 eV to 0.2 eV for 3d-band metals while may U vary for example by 2 eV, the practical effect of J in our calculations is secondary compared to U, which is agreement with the above given discussion.

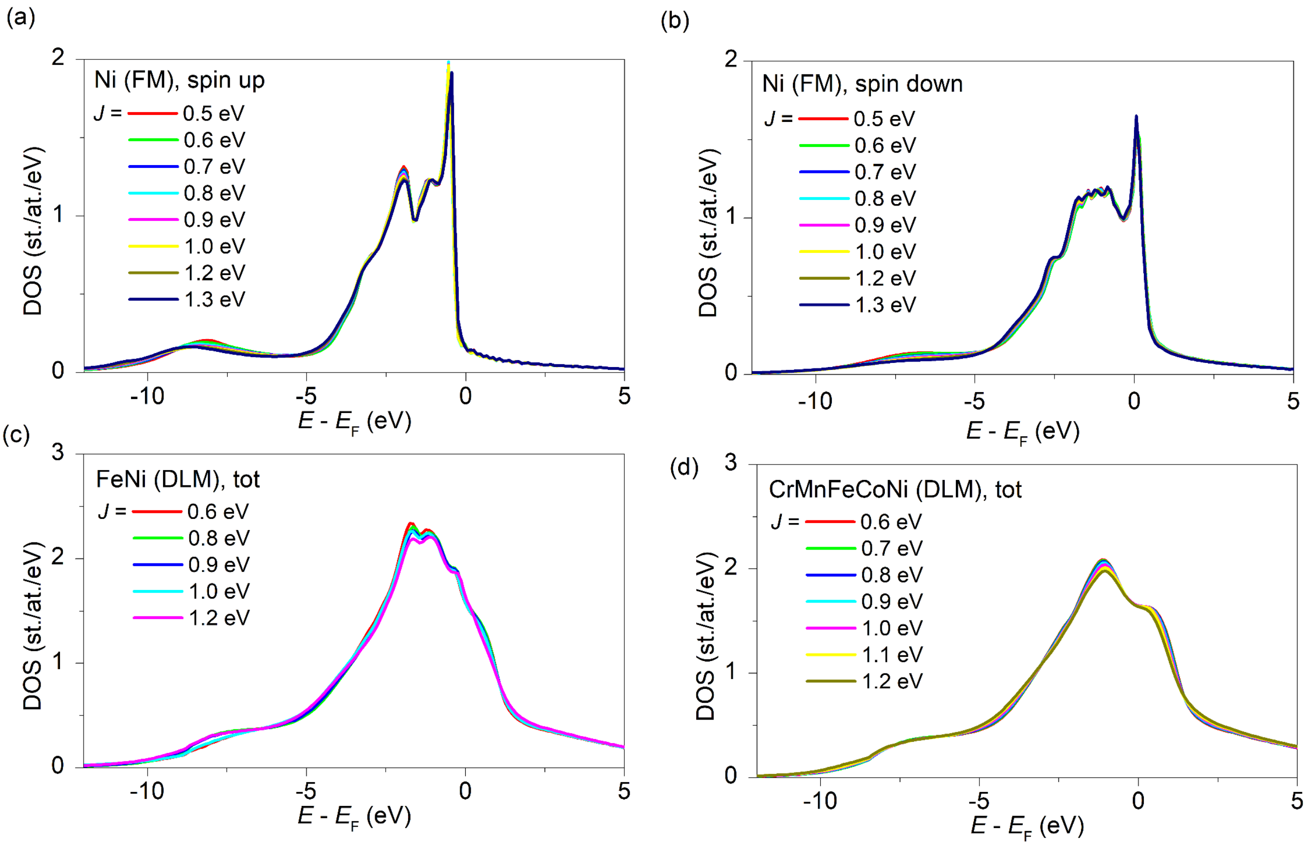

Supplementary Fig. 3 Density of States (DOS) with varying J values, ranging from 0.5 eV to 1.3 eV, for different magnetic configurations and compositions. The Coulomb interaction U is kept constant for all plots. (a) DOS for ferromagnetic (FM) Ni with spin up states. The satellite peak shifts from -8.2 eV (J = 0.5 eV) to -8.7 eV (1.3 eV) without notably altering the spectra between E_F and -5 eV. (b) DOS for FM Ni with spin down states. The shoulder-like feature gets smeared with increasing J. (c) Total DOS for paramagnetic FeNi, revealing a slight variation in the shoulder feature with increasing J. (d) Total DOS for CrMnFeCoNi. The shoulder remains unaffected, and there is no significant influence on the total spectra, solely a marginal shift of the spectral weight of the d-band towards higher binding energies with increasing J.

Supplementary Note 4: X-Ray absorption spectroscopy measurements

Supplementary Fig. 4 shows the wide scan X-Ray absorption spectroscopy measurements of CrMnFeCoNi for identifying the element specific L_3 absorption edges. Incident photon energy was 1200 eV. Experimental methods are described in the main manuscript.

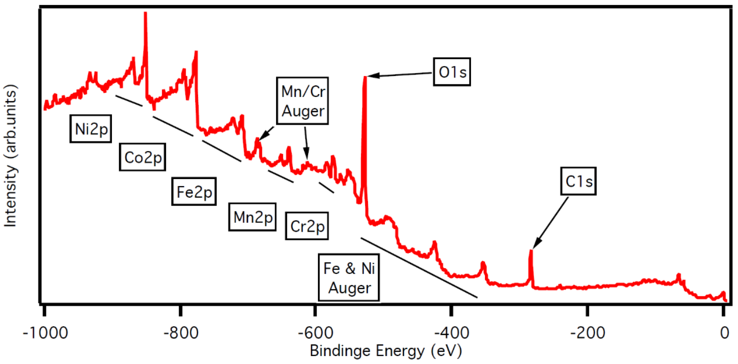

Supplementary Fig. 4 Wide scan X-Ray absorption spectroscopy measurements of CrMnFeCoNi, displaying intensity (arbitrary units) as a function of binding energy (eV). The spectrum reveals distinct peaks corresponding to core levels of various elements present in the sample. Peaks are identified for Ni 2p, Co 2p, Fe 2p, Mn 2p, and Cr 2p. Additionally, Auger peaks for Mn/Cr and Fe/Ni are observed. Prominent peaks at lower binding energies correspond to O 1s and C 1s, indicating the presence of oxygen and carbon on the sample surface. The annotations highlight the positions of these peaks.

Supplementary Note 5: Investigating element specific on-site Coulomb interaction within the Cini-Sawatzky Theory

Supplementary Fig. 5 delineates the calculated *d*-band pDOS of the constituent elements (Cr, Mn, Fe, Co, Ni) in yellow for the LDA and blue for the LDA+DMFT case, with the self-convolution on the two-particle energy scale depicted as dashed lines. The experimental data are represented by black solid lines. All curves are normalized to their respective maxima to simplify the analysis. In the bottom row in green results for an increased *U* for Fe and Ni from 1.5 eV to 2 eV and 3 eV to 4 eV, respectively are given. It may be clearly seen, that with variation of element specific *U* solely the concerned element is affected.

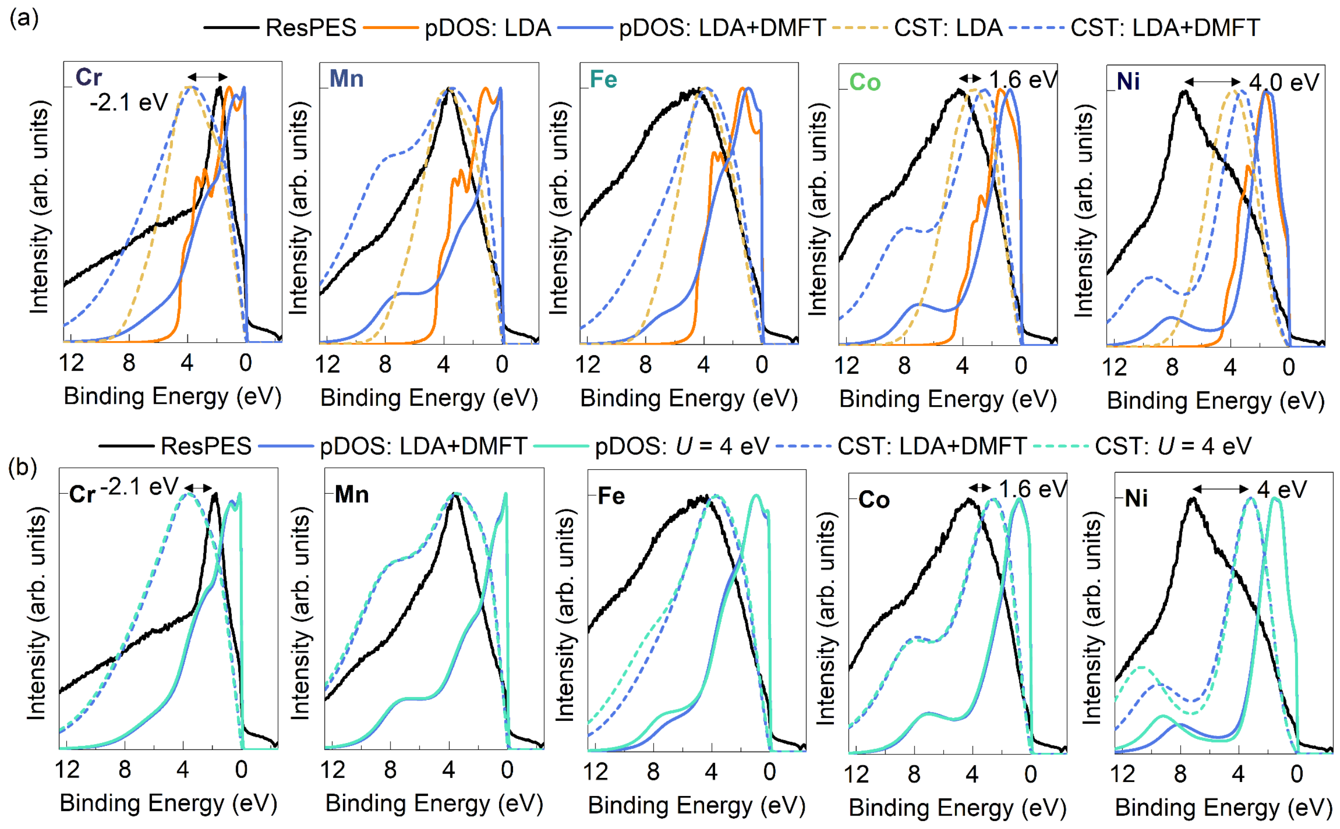

Supplementary Fig. 5 Application of the Cini-Sawatzky Theory (CST) by self-convolution of element resolved partial *d*-band DOS. Yellow line is for LDA and blue line for LDA+DMFT. Solid lines represent the pDOS and dashed lines the self-convoluted signal (plotted against 2 particle binding energy). Black line is the ResPES measurement at the XAS L_3 maximum. Arrows indicate the energetic difference between the peaks of the self-convoluted pDOS and the ResPES signal. (a) Calculations for LDA and *U* values as described above. (b) Additional calculation for varied *U* values for Fe = 2 eV and Ni = 4 eV.

Supplementary Note 6: Complex self-energy within the DMFT scheme

The complex self-energy was evaluated on the real energy axis, as going into the complex energy plane includes artificial damping effect, especially visible in the vicinity of the Fermi edge. This is demonstrated in Supplementary Fig. 6 for pure Ni calculations with J = 0.94 eV and U = 3 eV in the natural fcc crystal structure at room temperature. It may be clearly seen, that by evaluating the Greens function further away from the real axis, additional damping effects are included, which in turn reduce the lifetime, especially close to the Fermi edge.

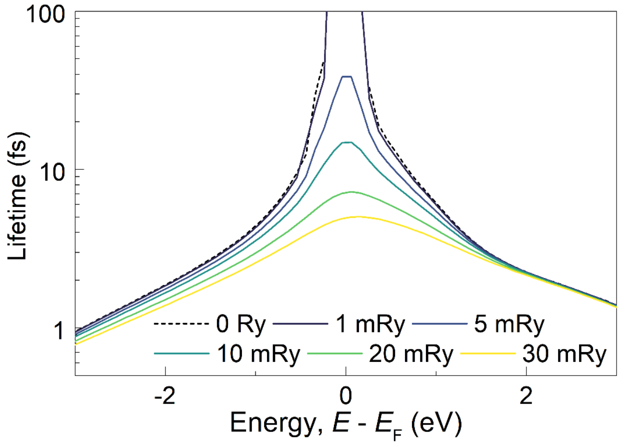

Supplementary Fig. 6 LDA+DMFT derived quasiparticle lifetimes for pure fcc Ni. Lines represent different complex energy shifts into the imaginary plane during evaluation of the imaginary part of the self-energy. Shift into imaginary plane is given in units of Ry.

As described in the main manuscript the influence of chemical disorder, structural changes impurities and eventually varying U values are investigated. Thus several subset of pure elements (black line) were calculated within their natural crystal structure and compared to pure elements within the fcc structure and lattice constant of the CrMnFeCoNi HEA (yellow line), as depicted in Supplementary Fig. 7(b). Thus the influence of structural changes may be investigates. Supplementary Fig. 7(a) shows the comparison on the pure elements on the fcc HEA crystal compared to the LDA+DMFT CPA potential for CrMnFeCoNi (blue line). Additionally the latter was varied with changing U values for Fe = 2 eV and Ni = 4 eV to test the influence of changing Hubbard U. We find that only these elements are affected by U variation and additionally the changes in lifetimes correspond to the fraction of old and new U values. A more detailed discussion is found in the main manuscript.

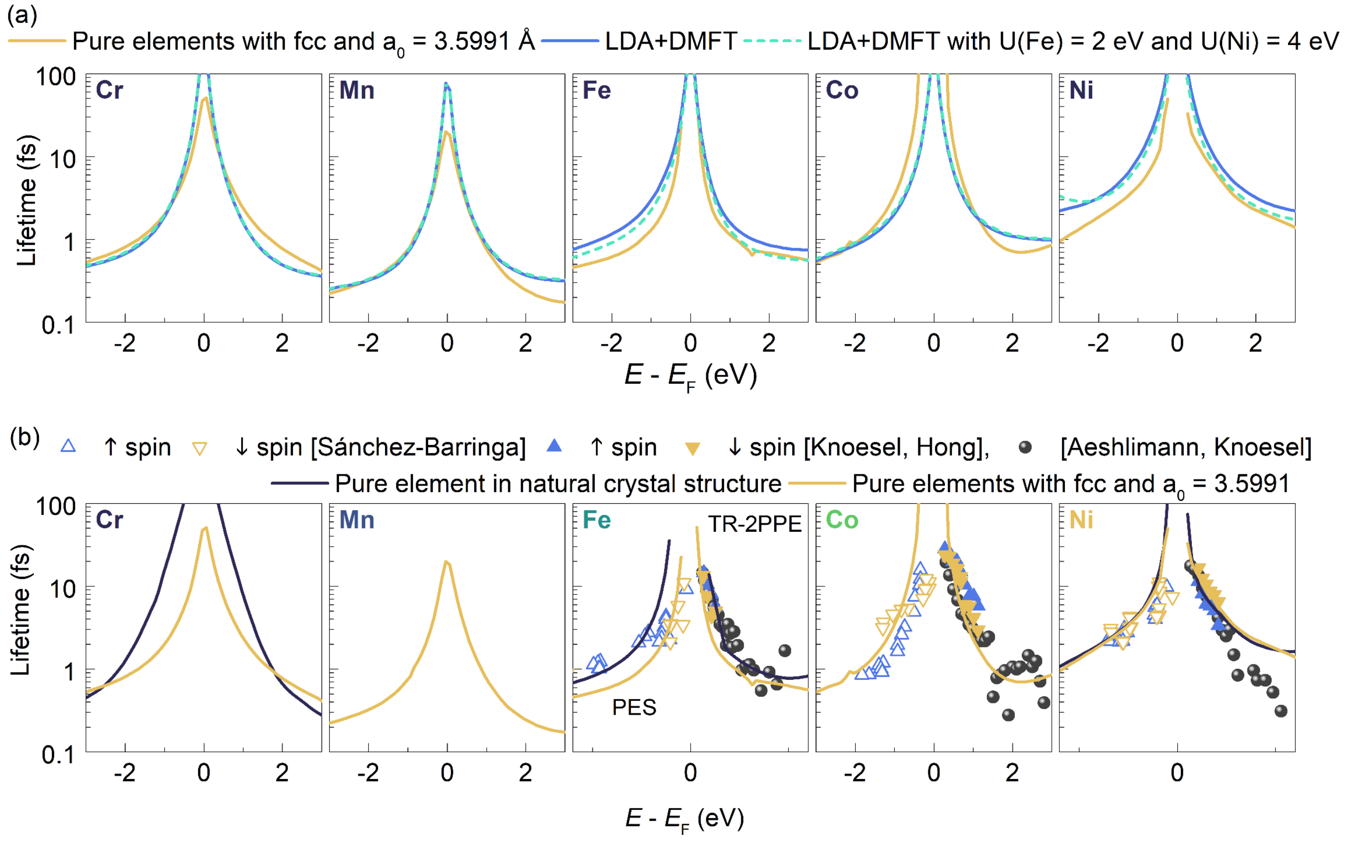

Supplementary Fig. 7 LDA+DMFT derived element specific quasiparticle lifetimes. (a) Yellow lines represent a pure metal case within the fcc structure and CrMnFeCoNi lattice constant. Blue lines represent the LDA+DMFT calculations of CrMnFeCoNi and green dashed lines LDA+DMFT calculations of CrMnFeCoNi with changed U values: Fe = 2 eV and Ni = 4 eV. (b) Comparison of experiments with pure element calculation in natural crystal (black line). Yellow lines are the same as in (a). Experimental data for pure elements are taken from Ref.[13] (below E_F) and Ref. [17–19] (above E_F).

Supplementary Note 7: Calculation of spectroscopic and transport properties

Valence band photoemission (PES) calculations were performed via the one-step model of photoemission, implemented in the SPR-KKR code[20].

Electronic transport as well the optical conductivity tensor where calculated within a linear response Kubo-framework. First the electrical resistivity was calculated taking into account electron-phonon collisions with increasing mean squared displacement of atomic positions with temperature within the alloy analogy model[21]. Hereby a large number of k-points in the reducible part of the BZ during integration of the current-current correlation function were used with 160000 k-points to ensure convergence. Within the alloy analogy model 30 temperatures ranging from 0 K up to 1200 K linearly distributed where calculated, while 14 possible independent directions for atomic oscillations were taken into account. Due to the displacement operator, and projection of spherical harmonics away from its origin, a higher number, which is in principle temperature dependent, of angular momentum expansion was used. Here we set $l_{\max} = 4$, which is enough for the investigated range of temperatures.

The response to an external oscillating electric field was calculated by reformulating the current-current correlation function in the basis of the crystals Green's function[22]. Due to the fully-relativistic description of the current density operator j in SPR-KKR, within this approach, the Drude contribution to the dielectric function is inherently described[22,23]. The macroscopic optical conductivity tensor is then calculated from the non-local optical conductivity by double integration over space coordinates, and the optical conductivity is given by

$$\sigma_{\mu\nu}(\omega) = \frac{i\hbar}{\pi^2}\frac{1}{\Omega}\int_\Omega d^3r \int_\Omega d^3r' \int_{E_b}^{\infty} dE' \int_{E_b}^{\infty} dE\, \Theta_T(E-E_F)\Theta_T(E_F-E')$$
$$\times \left\{ \frac{\mathrm{tr}\left[j_\mu(\mathbf{r})\mathrm{Im}G^+(E')j_\nu(\mathbf{r}')\mathrm{Im}G^+(E)\right]}{(E'-E-i\Gamma)(\hbar\omega+E-E'+i\Gamma)} + \frac{\mathrm{tr}\left[j_\nu(\mathbf{r}')\mathrm{Im}G^+(E')j_\mu(\mathbf{r})\mathrm{Im}G^+(E)\right]}{(E'-E-i\Gamma)(\hbar\omega+E'-E+i\Gamma)} \right\}.$$

In our results we focus only on $\sigma_{xx}(\omega)$, which will be solely denoted as $\sigma(\omega)$ in the following, since the paramagnetic CrMnFe-CoNi HEA is isotropic. The energy integrals (E_b is the bottom of the valence band) are performed by exploiting the analytic

properties of the Green's function by replacing the integration on the real energy axis with a complex contour integration technique[24]. Hereby the Fermi function Θ_T gives rise to Matsubara poles in the complex energy plane, and the contour integral has to be evaluated between two poles. Thus, electron temperature effects are taken into account directly, while the dominant lattice electron-phonon scattering, especially in the low frequency limit, is included within Γ. Further details on the choice of Γ will be discussed below. The choice of poles is limited by the fact that in our method the integration has to be performed for complex parts of the energies below $2Nk_{\mathrm{B}}T < \Gamma$, with N the number of Matsubara poles enclosed by the complex energy path. For our calculations at 300 K we choose $N = 2$. Besides the incorporation of chemical disorder through CPA and positional disorder due to phonon oscillations at given temperature within the alloy analogy model, in our approach electron-phonon collision are not inherently considered. However, these may be mimicked by the choice of Γ. For the linear response calculation a high number of 7500 k-points in the irreducible BZ were used during integration. 1000 photon energies, linearly distributed, from 0 eV to 8 eV where calculated.

Supplementary References

1. Ebert, H., Ködderitzsch, D. & Minár, J. Calculating condensed matter properties using the KKR-Green's function method—recent developments and applications. *Reports on Progress in Physics* **74**, 096501 (2011).
2. Ebert, H. The Munich SPR-KKR package. *version 8.6* http://olymp.cup.uni-muenchen.de/ak/ebert/SPRKKR (2022).
3. Vosko, S. H., Wilk, L. & Nusair, M. Accurate spin-dependent electron liquid correlation energies for local spin density calculations: a critical analysis. *Canadian Journal of Physics* **58**, 1200–1211 (1980).
4. Faulkner, J. S. & Stocks, G. M. Calculating properties with the coherent-potential approximation. *Physical Review B* **21**, 3222–3244 (1980).
5. Ebert, H., Vernes, A. & Banhart, J. Relativistic bandstructure of disordered magnetic alloys. *Solid State Communications* **104**, 243–247 (1997).
6. Wang, S., Zhang, T., Hou, H. & Zhao, Y. The Magnetic, Electronic, and Thermodynamic Properties of High Entropy Alloy CrMnFeCoNi: A First-Principles Study. *Phys. Status Solidi B* **255**, 1800306 (2018).
7. Gyorffy, B. L., Pindor, A. J., Staunton, J., Stocks, G. M. & Winter, H. A first-principles theory of ferromagnetic phase transitions in metals. *Journal of Physics F: Metal Physics* **15**, 1337–1386 (1985).
8. Winter, J., Sotrop, J., Borek, S., Huber, H. P. & Minár, J. Temperature-dependent determination of electron heat capacity and electron-phonon coupling factor for Fe0.72Cr0.18Ni0.1. *Physical Review B* **93**, 1–8 (2016).
9. Mu, S. *et al.* Uncovering electron scattering mechanisms in NiFeCoCrMn derived concentrated solid solution and high entropy alloys. *npj Computational Materials* **5**, (2019).
10. Minar, J. Correlation effects in transition metals and their alloys studied using the fully self-consistent KKR-based LSDA + DMFT scheme. *Journal of Physics Condensed Matter* **23**, (2011).
11. Belozerov, A. S., Katanin, A. A. & Anisimov, V. I. Itinerant magnetism of chromium under pressure: a DFT+DMFT study. *J. Phys.: Condens. Matter* **33**, 385601 (2021).
12. Di Marco, I. *et al.* γ-Mn at the border between weak and strong correlations. *Eur. Phys. J. B* **72**, 473–478 (2009).
13. Sánchez-Barriga, J. *et al.* Effects of spin-dependent quasiparticle renormalization in Fe, Co, and Ni photoemission spectra:An experimental and theoretical study. *Physical Review B* **85**, 205109 (2012).
14. Woodgate, C. D., Hedlund, D., Lewis, L. H. & Staunton, J. B. Interplay between magnetism and short-range order in medium- and high-entropy alloys: CrCoNi, CrFeCoNi, and CrMnFeCoNi. *Phys. Rev. Materials* **7**, 053801 (2023).
15. Miyake, T. & Aryasetiawan, F. Screened Coulomb interaction in the maximally localized Wannier basis. *Phys. Rev. B* **77**, 085122 (2008).
16. Keshavarz, S., Schött, J., Millis, A. J. & Kvashnin, Y. O. Electronic structure, magnetism, and exchange integrals in transition-metal oxides: Role of the spin polarization of the functional in DFT+ U calculations. *Phys. Rev. B* **97**, 184404 (2018).
17. Knoesel, E., Hotzel, A. & Wolf, M. Temperature dependence of surface state lifetimes, dephasing rates and binding energies on Cu(111) studied with time-resolved photoemission. *Journal of Electron Spectroscopy and Related Phenomena* **88–91**, 577–584 (1998).
18. Hong, J. & Mills, D. L. Theory of the spin dependence of the inelastic mean free path of electrons in ferromagnetic metals: A model study. *Phys. Rev. B* **59**, 13840–13848 (1999).
19. Bauer, M., Marienfeld, A. & Aeschlimann, M. Hot electron lifetimes in metals probed by time-resolved two-photon photoemission. *Progress in Surface Science* **90**, 319–376 (2015).
20. Braun, J., Minár, J., Ebert, H., Katsnelson, M. I. & Lichtenstein, A. I. Spectral Function of Ferromagnetic 3 d Metals: A Self-Consistent LSDA + DMFT Approach Combined with the One-Step Model of Photoemission. *Phys. Rev. Lett.* **97**, 227601 (2006).
21. Ebert, H. *et al.* Calculating linear-response functions for finite temperatures on the basis of the alloy analogy model. *Phys. Rev. B* **91**, 165132 (2015).
22. Huhne, T. & Ebert, H. Fully relativistic description of the magneto-optical properties of arbitrary layered systems. *Phys. Rev. B* **60**, 12982–12989 (1999).
23. Ambrosch-Draxl, C. & Sofo, J. O. Linear optical properties of solids within the full-potential linearized augmented planewave method. *Computer Physics Communications* **175**, 1–14 (2006).
24. Huhne, T. Magneto-optical Kerr effect of multilayer and surface layer systems. *PhD Thesis, LMU Munich* (2000).
